# Effectiveness and Safety of Sacubitril/Valsartan in Patients with Chronic Kidney Disease—A Real-World Experience

**DOI:** 10.3390/jcm12041334

**Published:** 2023-02-07

**Authors:** Sara C. Pereira, Tiago Rodrigues, Afonso Nunes-Ferreira, João R. Agostinho, Fausto J. Pinto, Dulce Brito

**Affiliations:** 1 Heart and Vessels Department, Cardiology Division, Centro Hospitalar Universitário de Lisboa Norte, E.P.E., Av. Prof. Egas Moniz MB, 1649-028 Lisbon, Portugal; 2 Cardiovascular Centre of the University of Lisbon (CCUL), CAML, Lisbon School of Medicine, University of Lisbon, 1649-028 Lisbon, Portugal

**Keywords:** chronic kidney disease, glomerular filtration rate, heart failure with reduced ejection fraction, angiotensin receptor neprilysin inhibitor, sacubitril/valsartan

## Abstract

Background: data regarding the effectiveness and safety of sacubitril/valsartan in heart failure and reduced ejection fraction (HFrEF) patients with chronic kidney disease (CKD) are scarse. Objective: to evaluate the effectiveness and safety of sacubitril/valsartan in HFrEF and CKD in a real-world population. Methods: we included consecutive ambulatory HFrEF patients that initiated sacubitril/valsartan between February 2017 and October 2020, stratified by CKD (KDIGO stage 5 excluded). Primary outcomes: the incidence rate per 100 patient-years and the annualized length of stay (LOS) of acute decompensated HF hospitalizations (HFH). Secondary outcomes: all-cause mortality, NYHA improvement, and titration of sacubitril/valsartan. Results: We included 179 patients, 77 with CKD, those being older (72 ± 10 vs. 65 ± 12 years, *p* < 0.001), had higher NT-proBNP (4623 ± 5266 vs. 1901 ± 1835 pg/mL, *p* < 0.001), and high anaemia incidence (*p* < 0.001). After 19 ± 11 months, a significant reduction in HFH adjusted incidence rate (57.5% decrease in CKD vs. 74.6%, *p* = 0.261) was observed, with 5 days there was a reduction in annualized LOS in both groups (*p* = 0.319). NYHA improved similarly in both groups (*p* = 0.670). CKD patients presented non-significant higher all-cause mortality (HR = 2.405, 95%CI: [0.841; 6.879], *p* = 0.102). Both groups had similar sacubitril/valsartan maximum dose achievement and drug withdrawal. Conclusion: sacubitril/valsartan was effective on reducing HFH and LOS without affecting all-cause mortality in a CKD real-world population.

## 1. Introduction

Patients with chronic kidney disease (CKD) present an increased risk of adverse cardiovascular events when compared with patients with normal renal function, including high rates of ischemic heart disease [1]. Additionally, patients with heart failure and reduced ejection fraction (HFrEF) are at a higher risk of adverse renal events due to reduced cardiac output, frequent episodes of acute decompensation with clinical congestion leading to aggressive diuretic therapy, and the risk of further glomerular damage [2,3]. Due to shared pathophysiology and risk factors (namely hypertension and diabetes mellitus), HF and CKD frequently coexist, carrying high morbidity and mortality [1,4,5].

The angiotensin receptor neprilysin inhibitor (ARNI) sacubitril/valsartan (S/V) was demonstrated in the Prospective Comparison of ARNI with ACEI to Determine Impact on Global Mortality and Morbidity in Heart Failure (PARADIGM-HF) trial to be superior to enalapril in reducing the risk of heart failure hospitalization and all-cause mortality [6]. The effect of S/V on cardiovascular morbidity and mortality was not modified by the baseline CKD status and fewer patients discontinued S/V because of renal impairment when compared with the enalapril group [6]. Furthermore, during follow up, the decline rate of the estimated glomerular filtration rate (eGFR) was slower in patients on S/V therapy compared with those on enalapril [7]. However, patients with estimated glomerular filtration rates (eGFR) of less than 30 mL/min/1.73 m^2^ were not included in the PARADIGM-HF trial and currently data regarding the safety and effectiveness of ARNI in these patients are scarce.

Some recent publications suggest that S/V in patients with CKD may offer great renal protection while maintaining beneficial effects on cardiovascular events, compared with renin-angiotensin-aldosterone system (RAAS) inhibition alone [8,9,10], supporting the beneficial effect of ARNI in HFrEF patients with CKD. As a result of the lack of data on the efficacy and tolerability of S/V in HFrEF patients with severe CKD, the current recommendation is to initiate S/V with caution in patients with eGFR < 30 mL/min/1.73 m^2^, starting at the lowest dose twice daily and not to use the drug in patients with end-stage renal disease (eGFR < 15 mL/min/1.73 m^2^) [11]. 

The purpose of this study was to evaluate the effectiveness of S/V in a real-world population of HFrEF patients with CKD in decreasing HF hospitalizations (HFH) and all-cause mortality, and in improving New York Heart Association (NYHA) functional class. For safety assessment, we evaluated S/V tolerability, titration, adverse effects, and withdrawal rates. 

## 2. Materials and Methods

### 2.1. Study Design and Population

We conducted a retrospective single-center study of consecutive HFrEF patients followed in a dedicated HF clinic (tertiary hospital), treated according to the recommendations of the European Society of Cardiology (ESC), that was initiated S/V between February 2017 and October 2020 [12].

The main inclusion criteria were (1) age >18 years, (2) diagnosis of HFrEF with left ventricular ejection fraction (LVEF) less than 40% documented by transthoracic echocardiography, and (3) symptomatic patients (NYHA functional class II–IV). The main exclusion criteria were (1) end-stage renal disease (KDIGO stage 5 classification [13]) including patients under dialysis, (2) moderate to severe chronic hepatic disease (Child–Pugh C class), (3) documented angiotensin receptor blocker (ARB) intolerance/allergy, and (4) Cardiac Resynchronization Therapy (CRT) during follow up after sacubitril/valsartan initiation. 

Patients were followed according to a standard protocol-based program for HF patients (Appendix A) [13]. For characterization and analysis, patients were divided in two groups according to renal impairment at baseline (before S/V initiation): eGFR ≥ 60 mL/min/1.73 m^2^ (patients without CKD) and eGFR < 60 mL/min/1.73 m^2^ (patients with CKD).

### 2.2. Data Collection and Outcomes

Demographic, clinical, [NYHA functional class, current medical therapy (drugs and devices)] and laboratory data at the time of S/V treatment initiation (baseline) and during follow-up were collected from medical records. Data regarding HFH were obtained 12-months prior and after S/V initiation (during the follow up period). Data on all-cause mortality were collected from medical records and national database. 

The primary outcomes were the incidence rate (per 100 patient-years) and annualized length of stay (LOS) of HFH. All-cause mortality, change in NYHA functional class, and titration of S/V during follow-up were secondary outcomes. Patient’s characteristics and outcomes were compared according to the presence or absence of renal impairment. In addition, a sub-analysis of CKD patients that initiated S/V with eGFR 15–29 mL/min/1.73 m^2^ at baseline was performed.

### 2.3. Statistical Analysis

Demographic, clinical, and therapeutic data at baseline were evaluated through measures of central tendency (mean) and dispersion (standard deviation: SD) for continuous variables with a normal distribution or median (interquartile range: IQR) for continuous variables with a non-normal distribution and absolute and relative frequencies (%) for categorical variables. 

Differences in continuous and categorical baseline variables between patients with and without CKD were evaluated with Wilcoxon signed-rank tests and chi-squared tests (or, whenever necessary, Fisher’s exact tests), respectively.

All study outcomes were analyzed comparatively for patients with and without CKD at baseline, adjusted for age, gender, NYHA functional class, LVEF, HF etiology, N-terminal-proB-type natriuretic peptide (NT-proBNP), and the comorbidities, with covariate analysis.

The effect of treatment initiation with S/V on incidence rate and annualized LOS of hospitalizations due to HF decompensation was analyzed using Poisson generalized linear mixed modeling with subject random effects and follow-up times as offsets. Differences between the resulting rate ratios (RR) for both groups were tested, including interactions between the treatment effect of S/V and CKD.

For survival analysis, Kaplan–Meier curves were used. The difference in overall survival between the two groups was tested by means of the log-rank test. Cox proportional hazards regression analysis was performed to estimate the hazard ratio of all-cause mortality between the two groups. 

Chi-squared tests (or Fisher’s exact tests) were further used to evaluate the differences in NYHA functional class changes and titration patterns of S/V during follow-up. Student T-test was used to compare numeric means of laboratorial data and LVEF across the same and different groups. When possible, 95% confidence intervals (CI) were reported and *p*-values < 0.05 were considered statistically significant. All statistical analyses were performed with the software R version 3.6 (R Foundation for Statistical Computing, Vienna, Austria) [14]. 

All patients included in this study freely signed an informed consent form (approved by the institutional ethics committee), authorizing prospective data collection for research purposes.

## 3. Results

### 3.1. Baseline Characteristics

A total of 179 HFrEF patients were included, and the mean follow-up period was 19 ± 11 months. The main HF etiologies were ischemic heart disease (52.5%) and dilated cardiomyopathy (36.3%). At baseline (before S/V initiation), 75% of patients presented in NYHA functional class II, 94% were taking a beta-blocker, and 70% and 69% of patients were taking an angiotensin-converting-enzyme inhibitor (ACEI) and mineralocorticoid receptor antagonist (MRA), respectively. CKD was identified in 77 patients (43%) with 11 (6.1%) having an eGFR of 15–29 mL/min/1.73 m^2^. Baseline demographic, clinical, and therapeutic characteristics, stratified by CKD status at baseline, are summarized in Table 1. Gender, NYHA functional class, LVEF, comorbidities, and concomitant drug and device therapies were similar between patients with and without CKD, except for the existence of anaemia (*p* = 0.014) and the use of diuretics (*p* = 0.002), which were both more prevalent in patients with CKD. Furthermore, patients with CKD were older (*p* < 0.001), less likely to be diagnosed with dilatated cardiomyopathy (*p* = 0.010), and had higher creatinine, urea, and NT-proBNP values than patients without CKD (*p* < 0.001 for all parameters). 

### 3.2. Hospitalizations Due to HF Decompensation

For both patients with and without CKD, treatment initiation with S/V resulted in a significant decrease in the incidence rate of hospitalizations due to HF decompensation during the follow up period compared with that observed prior to S/V therapy (Table 2). In patients with CKD the incidence rate estimates, adjusted for baseline demographic and clinical characteristics, decreased 57.5%, from 67.7 hospitalizations per 100 person-years before S/V treatment initiation, to 28.8 after S/V initiation (adjusted rate ratio (RR) = 0.425, 95% CI: [0.234, 0.773]). Despite an apparently higher reduction in incidence rate for patients without CKD, decreasing 74.6% from 64.0 hospitalizations per 100 person-years before S/V initiation, to 16.3 after S/V initiation (adjusted RR = 0.254, 95% CI: [0.133, 0.487]), it was not found to be significantly different from that estimated for patients with CKD (*p* = 0.261).

Significant relative reductions in the estimated annualized LOS after S/V treatment initiation were also similar in patients with CKD (Adjusted RR = 0.250, 95% CI: [0.164, 0.379]) and without CKD (Adjusted RR = 0.180, 95% CI: [0.112, 0.289], *p* = 0.319) (Table 2), resulting in an estimated absolute reduction of approximately 5 days per patient per year in both groups. 

### 3.3. All-Cause Mortality

**Figure 1:**
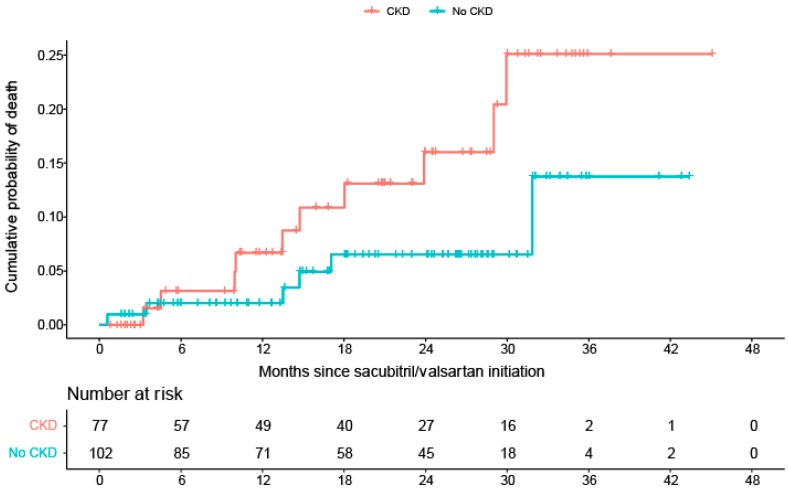
Kaplan–Meier Curves for all-cause mortality stratified by CKD at baseline. CKD: chronic kidney disease (eGFR < 60 mL/min/1.73 m^2^); eGFR: estimated glomerular filtration rate.

Among patients with CKD, ten patients (13%) died during the follow-up, compared with six (6%) without CKD. Although 24 months after S/V treatment initiation, the Kaplan–Meier estimated cumulative probability of all-cause mortality was 16% for patients with CKD and 6% for patients without CKD; no statistically significant difference in the cumulative all-cause mortality risk (*p* = 0.096, log-rank) was observed between the two groups over time (Figure 1).

The risk of all-cause mortality was estimated to be at least twice as high for patients with CKD than for patients without CKD, but without reaching statistical significance (adjusted hazard ratio (HR) = 2.405, 95% CI: [0.841; 6.879], *p* = 0.102).

### 3.4. NYHA functional class

A significant improvement was observed in NYHA functional class after ARNI initiation in both groups without significant differences (mean change from baseline (MCB) in CKD group = −0.53, 95% CI: [−0.67, −0.38]; without CKD group (MCB = −0.55, 95% CI: [−0.67, −0.43]; *p* = 0.670).

After follow-up, most patients were in NYHA functional classes I and II, with 28% of patients with CKD and 36% of patients without CKD reaching NYHA functional class I. Only one patient was at NYHA functional class IV (Table 3) after the follow up. NYHA class did not change for most patients (49%) in both groups (with and without CKD). Furthermore, in both groups 5% of patients improved by two NYHA functional classes. Only two patients, both with CKD at baseline, worsened by one NYHA functional class. 

No statistically significant differences were found in the NYHA functional class distribution, nor did the distribution of NHYA functional class change during follow up between patients with and without CKD at baseline (Table 3).

### 3.5. Sacubitril/Valsartan Titration

Irrespective of the presence of CKD at baseline, treatment with S/V was initiated at the lowest recommended starting dose (24/26 mg twice daily) in most of the patients in both groups (74% with CKD vs. 67% without CKD (*p* = 0.185, Table 4).

A maximum target dose of 97/103 mg twice daily was achieved during follow-up for 39% of patients with CKD and 33% of patients without CKD. Although a slightly higher percentage of patients without CKD were able to achieve a maximum dose of at least 49/51 mg twice daily during follow-up, no statistically significant differences in the distribution of the maximum achieved dose could be found when compared with patients with CKD (Table 4). There were no echocardiographic parameters at baseline that associated with higher S/V dose (49/51 mg bid or 97/103 mg bid) during follow up. 

At the last follow up visit, the number of patients at the target dose of 97/103 mg twice daily had decreased to 25% in both groups, due to dose reductions or withdrawal. Dose reductions were observed in 25% and 20% of patients with and without CKD, respectively (*p* = 0.390), whereas 17% of patients with CKD and 10% of patients without CKD withdrew sacubitril/valsartan (*p* = 0.151) (Supplementary Table S1). 

The main causes for dose reductions were symptomatic hypotension (that occurred in 56% of patients with CKD and in 61% of patients without CKD), followed by worsening kidney function (observed in 19% of patients with CKD) and decompensated heart failure (in 17% of patients without CKD). Dose interruptions were caused mainly due to hypotension (that occurred in 50% of patients with CKD and in 57% of patients without CKD), and economic constraints (in 17% of patients with CKD and in 29% of patients without CKD). Only one patient with CKD (stage 4 CKD, Supplementary Table S1) suspended S/V due to worsening renal function. During follow-up, none of the patients in either group required dialysis.

### 3.6. Subgroup Analysis of Patients with CKD Stage 4 at Baseline

In the studied cohort were included 11 patients with eGFR between 15 and 29 mL/min/1.73 m^2^ at baseline. The characteristics of these patients are detailed in Supplementary Table S2. The median age was 70 (IQR: 67–75) years, most patients were in NYHA functional class II (55%), and the most frequent etiology of HF was dilated cardiomyopathy (55%); the median LVEF was 28% (IQR: 18–31). Before S/V initiation, all patients were under triple therapy with ACEI or ARB, beta-blockers, and MRA. Most patients (82%) started S/V at the lowest recommended dose (Supplementary Table S3). During a median follow up period of 26 months (IQR: 2.3–36), the maximum dose of S/V (97/103 mg twice daily) was attained in three patients and the intermediate dose in another three patients. Five patients suspended S/V mainly due to hypotension (three patients), one patient due to cough, and another patient because of worsening kidney function, without requiring dialysis. There were no cases of drug interruption due to hyperkaliemia. Regarding the six patients that maintained S/V, five (83%) improved one NYHA functional class during the follow up period, but in the group of patients that interrupted S/V, only two (40%) patients improved symptoms. Two patients (out of 11) died during the follow up due to cardiovascular causes.

### 3.7. Effects of Sacubitril/Valsartan on Renal Function, Potassium Levels, NT-proBNP and Left Ventricular Ejection Fraction

From a total of 179 patients that initiated S/V, 155 (86.6%) patients maintained the drug during follow up, which is in line with previously described rates of S/V discontinuation in the literature [16,17]. The main reasons for withdrawal were already mentioned, hypotension being the most frequent (ten patients from the two groups). 

**Figure 2:**
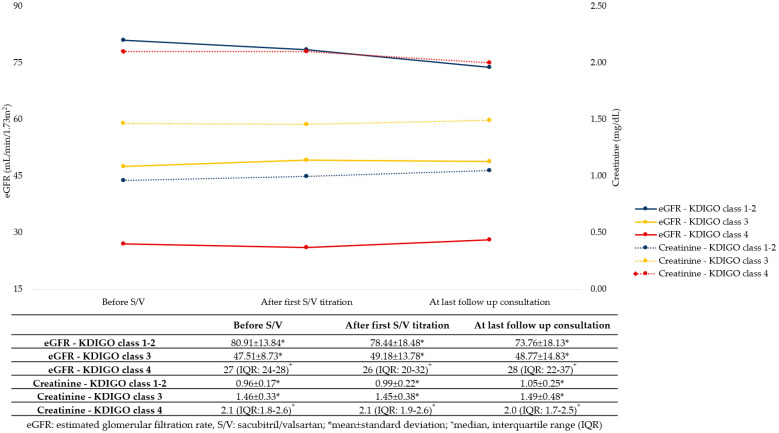
Creatinine and estimated glomerular filtration rate variation before, after first titration, and at last follow up consultation in patients that maintained sacubitril/valsartan at follow up.

Regarding renal function, a discrete and insignificant increase in serum creatinine was observed in patients with CKD stage 3 and in the non-CKD group at the last follow up consultation. However, despite the small group of patients, those in CKD stage 4 showed a tendency for creatinine stabilization or even a mild decrease during follow up (Figure 2). Potassium serum levels tended to be higher with S/V therapy as expected, and one patient (in CKD stage 3) reduced the S/V dose due to higher potassium levels, however none of the patients interrupted S/V therapy due to hyperkaliemia. 

The significant improvement observed in NYHA functional class with S/V therapy, previously detailed, was mirrored in patients with and without CKD by significantly lower levels of NT-proBNP (*p* < 0.001 for both groups) and improved LVEF (9.1 ± 11% vs. 8.1 ± 11%, *p* = 0.678) at the end of follow up, suggesting a positive remodeling benefit of the drug [18].

## 4. Discussion

This study showed that in a real-world HFrEF cohort of patients, the beneficial effects of S/V on hospitalizations, all-cause mortality, and clinical symptoms were not significantly influenced by the presence of CKD. Furthermore, CKD did not show to have a significant impact on S/V uptitration. This is in line with data from the PARADIGM-HF and related clinical trials, which have suggested that S/V offers greater renal protection compared with RAAS inhibition alone, while maintaining the beneficial effects on cardiovascular morbidity/mortality and clinical symptoms [7,8,9,10]. As opposed to most of these clinical trials, this study did not restrict the included population to patients with eGFR ≥ 30 mL/min/1.73 m^2^, but also included patients with eGFR of 15 to 29 mL/min/1.73 m^2^, in an attempt to add a bit more understanding on the effectiveness and tolerability of S/V in HFrEF patients with severe CKD.

The estimated incidence of hospitalizations due to HF decompensation dropped at least 55% after S/V initiation (57.5% in patients with CKD, 74.6% in patients without CKD), and the estimated annualized LOS decreased at least 75% (75.0% for patients with CKD, 82.0% for patients without CKD). A 10% difference in all-cause mortality at 24 months after S/V treatment initiation was estimated between patients with and without CKD (16% for patients with CKD and 6% for patients without CKD). Most patients either improved their NYHA functional class (49% and 51% of patients with and without CKD, respectively) or maintained their NYHA functional class unchanged (49% in both groups), leading to statistically significant improvements in NYHA functional class over a mean follow-up of 19 months for both groups. 

A recently published study regarding the high dose tolerability of S/V in a cohort of patients with HFrEF found that right ventricle (RV) dysfunction (TAPSE < 16 mm) plays a role in predicting S/V maximum dose tolerability [19]. In this study, the percentage of patients reaching a maximum S/V dose of 49/51 mg or 97/103 mg bid was 70% for patients with CKD and 80% for patients without CKD. However, across all populations of HFrEF patients, an initial higher TAPSE did not associate with a higher S/V dose during follow up. This finding may be explained by a normal mean baseline TAPSE across the two groups, exposing a global population with a preserved RV function, having less impact in S/V titration.

Patients with CKD also presented with other baseline characteristics typically associated with a worse prognosis: older age, more likely to be diagnosed with ischemic HF etiology, a higher prevalence of anaemia (probably due to CKD [20]) and higher NT-proBNP [21,22,23] albeit the fact that LVEF and functional class were similar between patients with or without CKD. This characteristic of worse prognosis might explain why patients with CKD presented with a lower relative reduction in HF-hospitalization rates and annualized LOS, higher all-cause mortality, and a slightly lower improvement in NYHA functional class. However, the fact is that the population with moderate to severe CKD showed to benefit with S/V therapy as well, and with no further deterioration on kidney function during the follow up time considered.

A few studies addressed the effectiveness and safety of S/V in CKD patients, with results similar to this study. A small study with 25 patients with advanced CKD (stage 3b and 4) showed that S/V improved LVEF after one year of follow up and reduced the number of visits to the emergency department due to HF decompensation [24]. Another study that included 52 patients with eGFR < 90 mL/min/1.73 m^2^, 30 with CKD (<60 mL/min/1.73 m^2^), had similar results regarding the improvement of LVEF, without significant variation of eGFR after one year of follow up, although the authors did not perform an analysis regarding clinical outcomes in HF hospitalizations [25]. Chang, et al. in a publication regarding a real-word cohort of HFrEF patients showed that S/V, when compared with previous standard HFrEF medical treatment, significantly reduced the composite endpoint of cardiovascular death or HF hospitalization in patients with significant renal function impairment at baseline (28% reduction over standard HF treatment in patients with eGFR < 30 mL/min/1.73 m^2^ vs. 14% reduction over standard HF treatment in patients with eGFR ≥ 30 mL/min/1.73 m^2^), although no stratified analyses on the effect of S/V on all-cause mortality or HF hospitalizations alone were performed [26].

Some small trials were conducted in patients with end-stage CKD under dialysis: Lee, et al. in a study with 23 HFrEF patients under dialysis, S/V on top of beta-blocker, and ivabradine significantly improved LVEF (23% variation), reduced high-sensitive troponin T, and soluble ST2 levels after a median follow up of 132 days [27]. A more recent case-control study compared 26 HFrEF patients with end-stage CKD treated with S/V vs. 23 patients under conventional therapy. The authors found that after one year of follow up, the patients under S/V significantly improved LVEF and diastolic function, with good tolerability, without significant differences regarding intradialytic hypotension and hyperkalemia. The hospitalization rate was similar between the two groups [28].

Real-world evidence concerning the effect of CKD on S/V titration was presented in an Italian study on 54 consecutive HFrEF patients (October 2016 to October 2017), initiating S/V [24]. Besides demonstrating that renal function improved more during the 12-month follow-up period in patients with CKD than in patients without CKD, this study also reported that S/V was more often up-titrated in patients with CKD (68% with a dose of 49/51 mg or 97/103 mg bid) than in patients without CKD (59% with a dose of 49/51 mg or 97/103 mg bid) [29].

Although our study considers unselected, sequential HFrEF patients initiating treatment with S/V within a broad spectrum of eGFR values, including patients with severe CKD (eGFR< 30 mL/min/1.73 m^2^), it has some limitations. Due to the observational, retrospective design, together with the lack of definition of a minimal follow-up period, heterogeneity was observed in the individual follow-up periods and also in the frequency of medical visits in different patients. Contributing to this aspect was the fact that part of the observational period of the study coincided with the COVID-19 pandemic, which, in Portugal, hampered the access to many healthcare services including medical appointments [30]. Further, due to the observational nature of this study, an imbalance in baseline characteristics between the groups of patients with and without CKD was observed, with a potential worse prognosis at baseline for patients with CKD. However, when possible, comparisons between the study groups were controlled for age, gender, NYHA functional class, LVEF, etiology of HF, NT-proBNP, and comorbidities by multivariate analysis. Additionally, as a limitation, this study has a small sample size, restricting statistical power. A larger study comparing patients with CKD 3a, 3b, and 4 would be helpful to address the safety and effectiveness of S/V more accurately.

## 5. Conclusions

Sacubitril/valsartan can be titrated as safely and effectively in HFrEF patients with CKD as in patients without CKD, ultimately improving the prognosis of patients in terms of hospitalizations due to heart failure decompensation, and heart failure functional symptom burden, with no significant impact in all-cause mortality. Sacubitril/valsartan should integrate the therapy of patients with HFrEF even in the presence of moderate to severe kidney disease. However, the monitoring of renal function and kalaemia is needed, as this population, due to age and comorbidities, always has a major risk of cardiorenal events.

## Figures and Tables

**Table 1:** Demographic, clinical, and therapeutic characteristics of patients at baseline (before S/V therapy).

Baseline Characteristic	CKD (n = 77)	No CKD (n = 102)	*p*-Value
Age (years), mean ± SD	72 ± 10	65 ± 12	<0.001
Male gender, n (%)	56 (73)	78 (76)	0.568
LVEF (%), mean ± SD	27 ± 6	28 ± 8	0.507
LV end-diastolic volume (mL)	202 ± 115	195 ± 85	0.495
LV end-systolic volume (mL)	145 ± 90	143 ± 76	0.782
TAPSE (mm), mean ± SD	18.7 ± 4.6	20.1 ± 4.7	0.997
NYHA functional class, n (%)			0.106
II	53 (69)	81 (79)	
III	24 (31)	21 (21)	
eGFR (mL/min/1.73 m^2^), mean ± SD	44.05 ± 11.50	81.19 ± 13.82	
eGFR ≥90 mL/min/1.73 m^2^, n (%)	-	30 (29)	
eGFR 60–89 mL/min/1.73 m^2^, n (%)	-	72 (71)	
eGFR 30–59 mL/min/1.73 m^2^, n (%)	66 (86)	-	
eGFR 15–29 mL/min/1.73 m^2^, n (%)	11 (14)	-	
Etiology of HF, n (%)			0.010
Ischemic CMP	43 (57)	51 (50)	
Dilatated CMP	20 (26)	45 (44)	
Other	13 (17)	6 (6)	
Comorbidities, n (%)			
Hypertension	48 (62)	64 (63)	0.956
Diabetes mellitus	41 (53)	41 (40)	0.083
Dyslipidaemia	53 (69)	63 (62)	0.327
Anaemia	21 (27)	13 (13)	0.014
COPD	17 (22)	24 (24)	0.819
Atrial fibrillation	39 (51)	38 (37)	0.073
Creatinine (mg/dL), mean ± SD	1.59 ± 0.49	0.95 ± 0.17	<0.001
Urea (mg/dL), mean ± SD	77 ± 38	46 ± 18	<0.001
Potassium (mmol/L), mean ± SD	4.61 ± 0.54	4.51 ± 0.50	0.316
NT-proBNP (pg/mL), mean ± SD	4623 ± 5266	1901 ± 1835	<0.001
SBP (mmHg), mean ± SD	118 ± 17	120 ± 18	0.604
Concomitant drug/device therapy			
ACEI, n (%)	52 (68)	73 (72)	0.493
ARB, n (%)	17 (22)	22 (22)	0.962
Beta-blocker, n (%)	72 (94)	96 (94)	>0.99
MRA, n (%)	49 (64)	74 (73)	0.203
Ivabradine, n (%)	15 (19)	18 (18)	0.750
Diuretic, n (%)	66 (86)	66 (65)	0.002
On triple therapy ^a^, n (%)	43 (56)	67 (66)	0.222
ICD, n (%)	28 (36)	35 (34)	0.776
CRT ^b^, n (%)	21 (27)	24 (24)	0.568

ACEI: angiotensin converting enzyme inhibitors; ARB: angiotensin receptor blockers; CMP: cardiomyopathy; CKD: chronic kidney disease (eGFR < 60 mL/min/1.73 m^2^, calculated by the Chronic Kidney Disease Epidemiology Collaboration formula (CKD-EPI) [15]; COPD: chronic obstructive pulmonary disease; CRT: cardiac resynchronization therapy; eGFR: estimated glomerular filtration rate; HF: heart failure; ICD: implantable cardioverter defibrillator; LV: left ventricle; LVEF: left ventricular ejection fraction; MRA: mineralocorticoid receptor antagonists; NT-proBNP: N-terminal-proB-type natriuretic peptide; NYHA: New York Heart Association; SBP: Systolic blood pressure; SD: standard deviation; S/V: sacubitril/valsartan; TAPSE: tricuspid annulus plane systolic excursion ^a^ triple therapy included ACEi/ARB, beta-blocker and MRA. ^b^ in the CKD group, 2 patients had a CRT implanted in the previous 6 months before the inclusion vs. 4 patients in the no CKD group (*p* = 0.495).

**Table 2:** Effect of treatment initiation with Sacubitril/Valsartan on the incidence rate and annualized LOS of hospitalizations due to HF decompensation stratified by CKD at baseline.

	CKD (n = 77)			No CKD (n = 102)			*p*-Value
	S/V		RR (95% CI)	S/V		RR (95% CI)	
	Before	After		Before	After		
**Incidence rate (per 100 person-years)**							
Crude ^a^	77.9	31.9	0.410 (0.216, 0.777)	66.3	16.3	0.246 (0.122, 0.495)	0.296
Adjusted ^b^	67.7	28.8	0.425 (0.234, 0.773)	64.0	16.3	0.254 (0.133, 0.487)	0.261
**Annualized LOS (days/year)**							
Crude ^a^	7.3	1.8	0.246 (0.161, 0.377)	6.0	1.1	0.178 (0.110, 0.287)	0.332
Adjusted ^c^	6.5	1.6	0.250 (0.164, 0.379)	5.9	1.1	0.180 (0.112, 0.289)	0.319

CKD: chronic kidney disease (eGFR < 60 mL/min/1.73 m^2^); eGFR: estimated glomerular filtration rate; HF: heart failure; LOS: length of stay; RR: rate ratio; S/V: sacubitril/valsartan; ^a^ Model with treatment, CKD and interaction; ^b^ Model with treatment, CKD and interaction, and adjusted for age, sex, NYHA, and anemia (final model); ^c^ Model with treatment, CKD and interaction, and adjusted for age, sex, ejection fraction, NYHA, and anemia (final model).

**Table 3:** Effect of treatment initiation with Sacubitril/Valsartan on NYHA functional class stratified by CKD at baseline.

	CKD (n = 76 ^a^)	No CKD (n = 100 ^a^)	*p*-Value
NYHA functional class at follow up, n (%)			0.137
I	21 (28)	36 (36)	
II	50 (66)	63 (63)	
III	4 (5)	1 (1)	
IV	1 (1)	0 (0)	
NYHA functional class change from baseline to last follow-up, n (%)			0.544
Improvement by 2 classes	4 (5)	5 (5)	
Improvement by 1 class	33 (43)	46 (46)	
Unchanged	37 (49)	49 (49)	
Worsened by 1 class	2 (3)	0 (0)	

CKD: chronic kidney disease (eGFR < 60 mL/min/1.73 m^2^); eGFR: estimated glomerular filtration rate; NYHA: New York Heart Association; ^a^ Missing values on NYHA functional class during follow-up: one patient with CKD, two patients with no CKD.

**Table 4:** Sacubitril/Valsartan dose titration stratified by CKD at baseline.

Sacubitril/Valsartan	CKD N = 77	No CKD N = 102	*p*-Value
Baseline dose, n (%)			0.185
24/26 mg bid	57 (74)	68 (67)	
49/51 mg bid	19 (25)	34 (33)	
97/103 mg bid	1 (1)	0 (0)	
Maximum dose achieved ^a^, n (%)			0.061
24/26 mg bid	23 (30)	20 (20)	
49/51 mg bid	23 (30)	48 (47)	
97/103 mg bid	30 (39)	34 (33)	
Dose at last follow-up, n (%)			0.250
24/26 mg bid	23 (30)	23 (23)	
49/51 mg bid	22 (29)	42 (41)	
97/103 mg bid	19 (25)	26 (25)	
Discontinued	13 (17)	11 (11)	

CKD: chronic kidney disease (eGFR < 60 mL/min/1.73 m^2^); eGFR: estimated glomerular filtration rate; bid: twice daily; ^a^ Missing values on NYHA functional class during follow-up: one patient with CKD.

## Data Availability

The data presented in this study are available on request from the corresponding author.

## Supplementary Materials

The following supporting information can be downloaded at: https://www.mdpi.com/article/10.3390/jcm12041334/s1, Table S1: Sacubitril/valsartan dosage and dose reduction/interruption; Table S2: Baseline characteristics of chronic kidney disease stage 4 patients; Table S3: Sacubitril/Valsartan dose titration in chronic kidney disease stage 4 patients.

## Appendix A. Protocol-Based Follow Up of Heart Failure Patients

- Patients admitted with acute decompensated heart failure in the Cardiology ward: routine visits at 7–10 days after discharge, followed by visits at one, three, six, and twelve months after discharge (and additionally, whenever considered necessary), with pre-specified procedures*.
- Patients admitted in the HF clinic due to chronic HF: routine visits after the first consultation, being the subsequent visit usually 2 weeks after the first evaluation, and then at the first, third, sixth, and twelfth month (and additionally, whenever considered necessary), with pre-specified procedures*. Patient’s education beginning during hospitalisation and maintained thereafter during follow up.

* Pre-specified procedures include:

(a) Clinical assessment aimed at identifying signs or symptoms of HF decompensation, residual congestion, or low cardiac output;
(b) Laboratory assessment, including monitoring of plasma N-terminal pro-brain natriuretic peptide (NT-proBNP) value, end-organ dysfunction, and development of common comorbidities in HF patients (diabetes, chronic pulmonary disease, dyslipidemia, thyroid dysfunction, anemia, iron deficiency);
(c) Electrocardiogram at every visit, and transthoracic echocardiogram between the third and sixth month after discharge and every twelve months of follow-up; when considered necessary, cardiac magnetic resonance imaging study is requested, as are other instrumental procedures;
(d) Assessment of adherence and tolerance to therapy;
(e) Individualized titration of therapy in accordance with the ESC guidelines;
(f) Patient education regarding self-care, lifestyle modifications, and management of HF decompensation.
