# Supplementary material for: Effectiveness and Safety of Sacubitril/Valsartan in Patients with Chronic Kidney Disease—A Real-World Experience
Source: J Clin Med. 2023 Feb 7;12(4):1334. doi: 10.3390/jcm12041334 (PMC9967392; doi:10.3390/jcm12041334)
Supplement: Supplementary file 1 [file jcm-12-01334-s001.zip › jcm-2180029-supplementary.pdf]

SUPPLEMENTARY MATERIAL

Effectiveness and safety of sacubitril/valsartan in patients with chronic kidney disease – a real-world experience

Authors: Sara Couto Pereira, Tiago Rodrigues, Afonso Nunes-Ferreira, João R. Agostinho, Fausto J. Pinto, Dulce Brito

Contents

Table S1. 2

Table S2. 3

Table S3 4

Table S1. Sacubitril/valsartan dosage and dose reduction/interruption

	eGFR 15-59 mL/min/1.73m^2 at baseline	eGFR ≥60 mL/min/1.73m^2 at baseline	p-value
**Dose reduction during follow-up, n (%)**	n=76	n=102	
	19 (25)	20 (20)	0.39
**Cause of dose reduction , n (%)**			0.4
Worsening kidney function	3 (16)	0	
Angioedema	0	1 (5)	
Decompensated heart failure	1 (5)	3 (15)	
Hyperkalaemia	1 (5)	0	
Hypotension	9 (47)	11 (55)	
Economic constraint	1 (5)	1 (5)	
Cough	1 (5)	2 (10)	
Unknown	3 (16)	2 (10)	
**Dose interruption during follow-up, n (%)**	n=76	n=102	
	13 (17)	10 (10)	0.15
**Cause of dose interruption , n (%)**			0.69
Worsening kidney function	1 (8)	0	
Angioedema	0	1 (10)	
Decompensated heart failure	2 (15)	0	
Hypotension	6 (46)	4 (40)	
Economic constraint	2 (15)	2 (20)	
Cough	1 (8)	0	
Unknown	1 (8)	3 (30)	

eGFR: estimated glomerular filtration rate

Table S2. Baseline characteristics of chronic kidney disease stage 4 patients

Baseline Characteristics			
**Age (years), median (IQR)**	70 (67-75)	**Laboratory results (median (IQR)**	
**Male gender, n (%)**	8 (73)	Creatinine (mg/dL)	2.37 (1.86-2.86)
**LVEF (%), median (IQR)**	28 (18-31)	Urea (mg/dL)	115 (100-181)
**NYHA functional class, n (%)**		Potassium (mmol/L)	5.0 (4.7-5.3)
**II**	6 (55)	NT-proBNP (pg/mL)	6273 (2644-12759)
**III**	5 (45)		
**eGFR (mL/min/1.73m2), mean ± SD**	24.8 ± 3.5 (18-29)	**SBP** (mmHg), median (IQR)	114 (102-130)
**Etiology of HF, n (%)**			
Ischemic CMP	4 (36)	**Concomitant drug/device therapy, n (%)**	
Dilatated CMP	6 (55)	ACEI	8 (73)
Other	1 (9)	ARB	2 (18)
**Comorbidities, n (%)**		Beta-blocker	11 (100)
Hypertension	5 (46)	MRA	7 (64)
Diabetes mellitus	6 (55)	Ivabradine	1 (9)
Dyslipidaemia	8 (73)	Diuretic	11 (100)
Anaemia	5 (46)	On triple therapy*	11 (100)
COPD	4 (36)	ICD	3 (27)
Atrial fibrillation	9 (82)	CRT	6 (55)

eGFR: estimated glomerular filtration rate; SD: standard deviation; LVEF: left ventricular ejection fraction; NYHA: New York Heart Association; HF: heart failure; CMP: cardiomyopathy; COPD: chronic obstructive pulmonary disease; SBP: systolic blood pressure; ACEI: angiotensin converting enzyme inhibitors; ARB: angiotensin receptor blockers; ICD: implantable cardioverter defibrillator; MRA: mineralocorticoid receptor antagonists; NT-proBNP: N-terminal-proB-type natriuretic peptide; S/V: sacubitril/valsartan
[a] triple therapy included ACEi/ARB, beta-blocker and MRA.

Table S3. Sacubitril/Valsartan dose titration in chronic kidney disease stage 4 patients

Sacubitril/Valsartan	**eGFR 15-29 mL/min/1.73m^2 n=11**
Baseline dose, n (%)	
24/26 mg bid	9 (82)
49/51 mg bid	2 (18)
97/103 mg bid	0
Maximum dose achieved[a], n (%)	
24/26 mg bid	5 (46)
49/51 mg bid	3 (27)
97/103 mg bid	3 (27)
Dose at last follow-up, n (%)	
24/26 mg bid	3 (27)
49/51 mg bid	2 (18)
97/103 mg bid	1 (9)
Discontinued	5 (46)

CKD: chronic kidney disease (eGFR < 60 mL/min/1.73m^2); eGFR: estimated glomerular filtration rate; bid: twice daily;
